# Seven decades towards malaria elimination in Yunnan, China

**DOI:** 10.1186/s12936-021-03672-8

**Published:** 2021-03-12

**Authors:** Xiao-Hong Li, Hong-Ning Zhou, Jian-Wei Xu, Zu-Rui Lin, Xiao-Dong Sun, Jia-Yin Li, Xian-Xian Lin, Yan Xie, Pedro Alonso, Heng-Lin Yang

**Affiliations:** 1 Global Malaria Programme, World Health Organization, Geneva, Switzerland; 2 Yunnan Institute of Parasitic Diseases, Yunnan Provincial Centre of Malaria Research, Yunnan Provincial Key Laboratory of Vector-Borne Disease Control and Research, Yunnan Provincial Collaborative Innovation Center for Public Health and Disease Prevention and Control, Pu’er City, China; 3 Yunnan Representative Office, Health Poverty Action (UK), Kunming, China; 4 School of Public Health, Peking University, Beijing, China

**Keywords:** Malaria elimination, Border malaria, Mass drug administration, Stratification

## Abstract

**Background:**

Yunnan Province was considered the most difficult place in China for malaria elimination because of its complex malaria epidemiology, heterogeneous ecological features, relatively modest economic development, and long, porous border with three malaria endemic countries: Lao People’s Democratic Republic, Myanmar, and Viet Nam.

**Methods:**

Academic publications and grey literature relevant to malaria elimination in Yunnan covering the period from 1950 until 2020 inclusive were considered. The following academic indexes were searched: China Science Periodical Database, China National Knowledge Infrastructure Database, and MEDLINE. Grey literature sources were mainly available from the National Institute of Parasitic Diseases (NIPD), the Chinese Center for Diseases Control and Prevention, and the Yunnan Institute of Parasitic Diseases (YIPD).

**Results:**

A malaria elimination campaign in the 1950–1960s, based mainly on mass administration of antimalarial drugs and large-scale vector control, reduced morbidity and mortality from malaria and interrupted transmission in some areas, although elimination was not achieved. Similar strategies were used to contain outbreaks and a resurgence of disease during the 1970s, when malaria services were discontinued. From the 1980s, malaria incidence declined, despite the challenges of large numbers of mobile and migrant populations and an unstable primary health care system in rural areas following economic transformation. Launch of the national malaria elimination programme in 2010 led to adoption of the ‘1–3-7′ surveillance and response strategy specifying timely detection of and response for every case, supported by the establishment of a real-time web-based disease surveillance system and a new primary health care system in rural areas. Border malaria was addressed in Yunnan by strengthening the surveillance system down to the lowest level, cross-border collaboration with neighbouring countries and non-governmental organizations, and the involvement of other sectors.

**Conclusions:**

Seven decades of work to eliminate malaria in Yunnan have shown the importance of political commitment, technically sound strategies with high quality implementation, a robust surveillance and response system at all levels, community participation and effective management of border malaria. The experiences and lessons learned from elimination remain important for prevention re-establishment of malaria transmission in the Province.

**Supplementary Information:**

The online version contains supplementary material available at 10.1186/s12936-021-03672-8.

## Background

Over many centuries, Yunnan Province in southwest China has suffered a heavy burden of disease, death, and social disruption from malaria. Documents from as early as 225 AD, indicate that malaria devastated the population of Yunnan. Between 1901 and 1907, many of the 60,000–70,000 workers who died during construction of the Yunnan and Viet Nam railway succumbed to malaria [1, 2]. One epidemic in Si’mao (now Pu’er) lasted from 1919 to 1932 and reduced the population of the city from 30,000 to 1000 [3]. More recently, over the last 30 years Yunnan consistently reported the highest number of malaria cases and deaths of any province in China [4]. The last indigenous cases of *Plasmodium falciparum* and *Plasmodium vivax* in Yunnan, registered in 2015 and 2016, respectively, were also the last indigenous cases of either species in China [5–8].

In the 1950–60 s, pioneering work in Yunnan during China’s malaria eradication programme succeeded in interrupting transmission in some areas, including places with a baseline prevalence exceeding 40% [2]. This legacy was largely documented in ‘grey literature’, including documents and reports by government and academics preserved in libraries and archives. These records represent a valuable knowledge repository, now that 50 years later, malaria elimination is once more affirmed as a national goal.

**Fig. 1:**
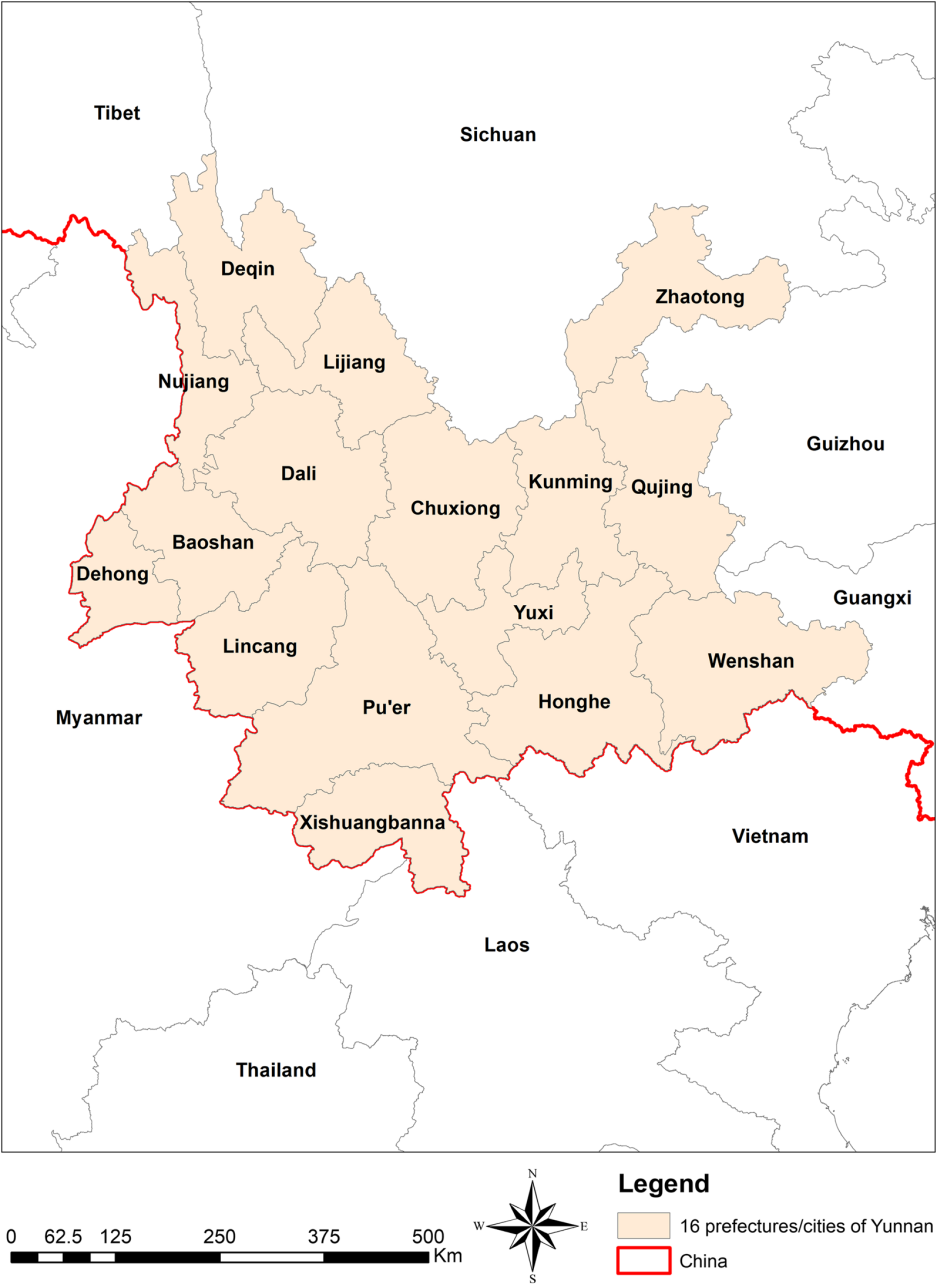
Geographic location of Yunnan Province, China. The map shows the location of Yunnan Province and its borders with Myanmar in the west, the Lao People’s Democratic Republic in the south-east and Viet Nam in the east. Within China, Yunnan borders Guangxi, Guizhou and Sichuan provinces and Tibet autonomous region. Yunnan is divided into 16 prefectures

Mirroring the path of China as a whole, malaria elimination in Yunnan has been accompanied by the evolution of the health system and social and economic development. However, the long and arduous journey to malaria elimination in Yunnan can be attributed to a complex malaria epidemiology, ecological heterogeneity, relatively modest economic development, and the 4000 km porous border with three malaria endemic countries, i.e. the Lao People’s Democratic Republic, Myanmar and Viet Nam (Fig. 1).

A malaria-free world remains the vision of the global community. One of the lessons learned from the Global Malaria Eradication Programme is that success depends on how and when malaria is eliminated from the most problematic regions. This article describes the complexity of malaria elimination in the context of Yunnan, reviews the strategies and interventions employed over the last seven decades, and considers the multifaceted nature of border malaria in Yunnan. Finally, the challenges for malaria elimination and preventing the re-establishment of transmission are discussed. Reconstructing the history of malaria elimination over the last seven decades in Yunnan, the most challenging region for malaria in China, will be informative to programmes and individuals currently engaged in malaria elimination, and may encourage those pursuing the dream of a malaria-free world.

## Methods

Academic publications and grey literature relevant to malaria elimination in Yunnan covering the period from 1950 until 2020 inclusive were considered. The following academic indexes were searched: China Science Periodical Database, China National Knowledge Infrastructure Database, and MEDLINE. Significant efforts were made to search grey literature sources, mainly available from the National Institute of Parasitic Diseases (NIPD) (the technical leader of the national malaria programme), and the Yunnan Institute of Parasitic Diseases (YIPD) (the technical leader of Yunnan malaria programme). The grey literature included a range of different documents, most of which were not published and only available in the archives: (1) books published in Chinese, authored by professors or senior technical officers associated with the NIPD and YIPD, regarding the history of malaria programmes in Yunnan and China, key implementation strategies and interventions, and their impact; (2) operational manuals or guidance for the selection and application of malaria interventions for control and elimination published by the Bureau of Disease Control of the Ministry of Health, the health authorities of the Government, NIPD, and YIPD; (3) technical strategies, action plans, drug policies, and regulations, issued by the Bureau of Disease Control of the Ministry of Health; (4) reports of pilot studies and projects, including the original records and work diaries of implementers, and the annals produced by YIPD which includes a summary of projects and pilot studies conducted by the YIPD from the 1950s to 1990s; (5) annual provincial programme review reports, planning reports, including annual planning and five-year action plans; (6) a report evaluating the implementation of malaria projects funded by the Global Fund to Fight AIDS, Tuberculosis and Malaria (Global Fund) in Yunnan, authored by the project manager; and (7) statistics published on the official website of the Government. Information was cross-checked to ensure validity. In case of discrepancy, reference was made to the original records if available. Additionally, key informants from the Province and country were interviewed to verify information or reconcile discrepancies.

### Malaria context in Yunnan

#### Geography and economic development

Yunnan has a population of 45 million (2010 sixth national census), divided into 16 prefectures and sub-divided into 129 counties (Fig. 1). It covers 394 000 km^2^ of largely mountainous terrain. Average altitude is 1980 m, ranging from 76.4 m in Red River Valley to 6,740 m at Diqing plateau. Six major river systems run through the Province, including the Lancang River (Mekong in Thai). Much of the Province lies within the subtropical highland or humid subtropical zone, with mild-to-warm winters and temperate summers, while in the tropical south temperatures regularly exceed 30 °C in the warmer half of the year. The rugged, vertical terrain has a wide range of flora and fauna and rich ecological and biological diversity. While Yunnan Province has lifted nearly 37 million people out of poverty over the past four decades, poverty prevalence is three times the national average. In 2017, the Province had the second lowest per capita GDP in China, shorter life expectancy, a relatively modest health system (based on the indicator of health professionals per 10,000 population), and a high illiteracy rate [9].

#### Health system and surveillance

Since the 1950s, China’s primary health care system has included both health prevention and provision of medical care. In rural areas, a cooperative medical system was developed with collective financing and public support from the People’s Commune, in which ‘barefoot doctors’ (farmers who received basic medical and paramedic training and worked in rural villages) were the backbone of the provision of free health care to villagers. From the 1980s, this system gradually disappeared with economic transformation [10]. It was replaced by the New Cooperative Medical System in 2003, providing health insurance to about 98.7% (802 million) of the rural population by 2013 [11].

Disease prevention and public health services were transformed after the SARS outbreak in 2003, including the creation of the Chinese Centre for Disease Control and Prevention (CDC). At the county level, the CDC is the most basic unit providing epidemiological and other public health services. Below the county level, disease prevention and control are generally managed by one or two qualified doctors based at the township hospital, and by one part-time doctor in villages. China has conducted surveillance for communicable diseases at disease surveillance points in counties since the 1950s. Malaria cases were recorded in monthly reports from counties, which were delivered by post until 1985; between 1985 and 2003, malaria cases were recorded monthly and reported electronically; and following the establishment of the CDC, real-time, case-specific, web-based disease surveillance was instituted.

#### Malaria epidemiology

**Fig. 2:**
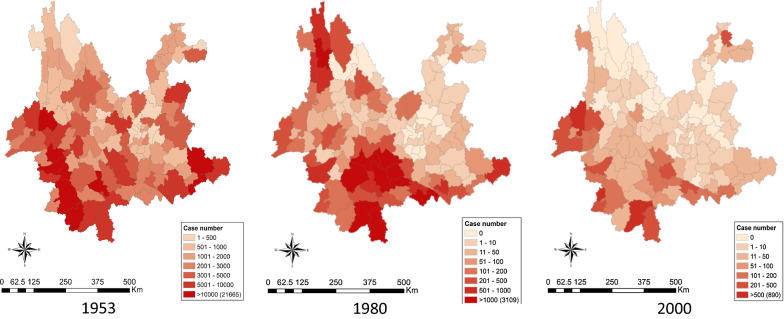
Spatial distribution of malaria cases in Yunnan. In 1953, cases were distributed throughout the Province but densely distributed in the south or south-west and in Wenshan in the south-east; in 1980, cases concentrated in the Yuan River valley, in the south and north-west; in 2000, cases were mostly seen in border regions

All four human plasmodia species have been transmitted in Yunnan. *Plasmodium malariae* disappeared in the early 1960s; *P. falciparum* was typically seen south of N 25°, while *P. vivax* was widely distributed and subspecies with short and long incubation periods co-existed [12, 13]. The geographical distribution of malaria was characterized by latitude, altitude and terrain, with a high prevalence in river valleys and basins in low latitude and low altitude areas, though transmission could occur above 2000 m (Fig. 2) [2, 14].

At least six species of anophelines (*Anopheles minimus*, *Anopheles sinensis*, *Anopheles kunmingensis*, *Anopheles dirus*, *Anopheles anthropophagus*, and *Anopheles jeyporiensis*) were confirmed as malaria vectors with importance in transmission in Yunnan [15]. Anopheline species distribution, population composition, and their role in malaria transmission is influenced by altitude, climate, vegetation, and other ecological factors. For example, *An. minimus* was the main vector south of N 25° at altitudes < 1500 m, where the climate is warm and humid, with *An. jeyporiensis* a secondary vector. *Anopheles anthropophagus* was distributed broadly but confirmed as a malaria vector only in the north-east. Moreover, the role of malaria vectors in transmission changed over time, owing to urbanization and the large-scale use of insecticides in agriculture [16].

### Malaria control and elimination from 1950s until 2020

**Fig. 3:**
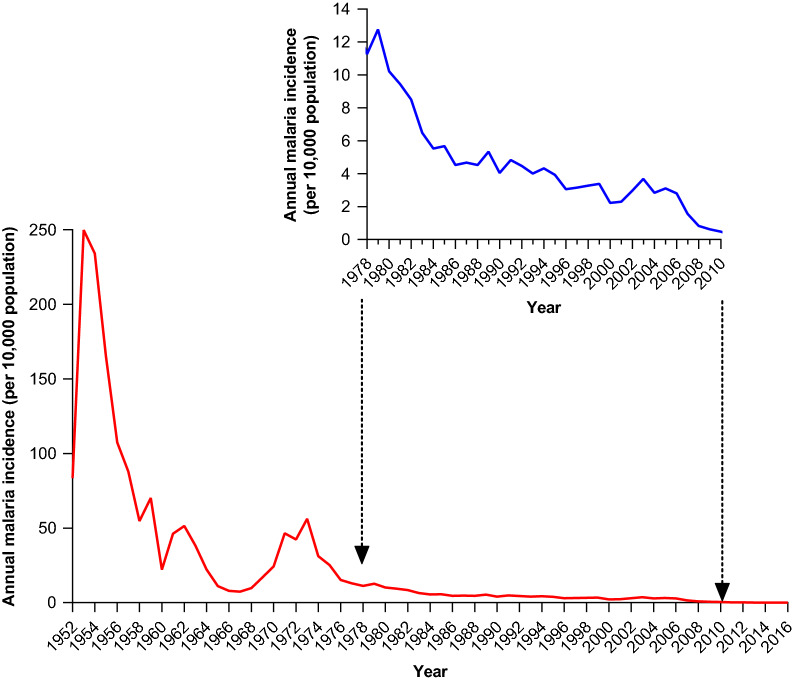
Malaria incidence in Yunnan 1952–2016 [2]. The inset shows data for 1978–2010 replotted on an expanded scale

The overall trend in malaria incidence was for a decrease from the 1950s, with the highest annual incidence of 249 per 10,000 population recorded in 1953. However, there was a significant resurgence in the 1960s–1970s, when the malaria service was discontinued during the Cultural Revolution. A subsequent decrease in malaria incidence during the 1980s, coincided with economic reform, and was sustained throughout the end of the twentieth century and into the twenty-first century (Fig. 3) [2].

#### Pursuing elimination to managing resurgence: 1950 to 1977

The goal of China’s first malaria control plan, launched in 1951, was to rapidly reduce morbidity and mortality from malaria in high transmission areas in the country, including parts of Yunnan [17]. Technical staff were deployed to Yunnan from all over the country, and 18 malaria stations were established to perform surveys, training, and pilot studies for gradual roll-out of control activities [18]. Epidemiological surveys and entomological investigations were extended throughout the Province after the country declared its ‘eradication’ goal in 1956. These disclosed significant heterogeneity for malaria transmission in Yunnan caused by latitude, altitude, terrain, and social determinants (Fig. 2). Consequently, all 129 counties were required to stratify villages into high, medium or low transmission on the basis of malaria prevalence or incidence [19]. The interventions, drugs and insecticides used changed rapidly, depending on the epidemiology, the response to the interventions, the results from pilot studies, and the availability of commodities and resources (Additional file 1: Table S1) [18, 20–28]. However, overall three basic strategies were employed: (1) clearance of the infection source; (2) chemoprevention, also called intermittent preventive treatment (IPT); and (3) vector control [18, 19, 29]. The intensity or frequency of implementation and the target population for each strategy differed by malaria transmission stratum (Table 1).

**Table 1:** Strategies for malaria elimination in Yunnan (1950–1970s)

Strategy, interventions	Malaria transmission stratum^a^	Timing and frequency
Clearance of the infection source		
Case management	High/medium/low: Clinical cases and laboratory-confirmed cases	Any time
Radical treatment	High: Individuals with a history of recent malaria; entire population or targeted population^b^	Any time for individuals; MDA during non-transmission seasons. Usually, one round MDA during non-transmission seasons. In high-transmission areas, several rounds of MDA might be necessary, before and during transmission seasons
	Medium/low: Individuals with a history of recent malaria; entire population living in active foci or targeted population^b^	
Chemoprevention		
Intermittent preventive treatment^c^	High: Entire population of villages and migrants	Start 1 month before the transmission season starts. Depending on the drugs used, application covered the entire transmission season; usually at least eight times per year
	Medium/low: Population in active foci, migrants	
Vector control		
Indoor residual insecticide spraying^d^	High: Houses and animal sheds in whole villages	During the transmission season. Depending on the duration of transmission seasons and the insecticide used, could be one, two or three rounds per year
	Medium/low: Houses and animal sheds in active foci	
Treatment of larval breeding sites	High: Use chemicals	At the end of winter and the beginning of spring
	Medium/low: Use whatever available (e.g. herbal)	

MDA, mass drug administration

^a^High transmission stratum: > 30% population with a history of malaria; annual incidence > 20%; incidence in children and new arrivals higher than that in adults; transmission of *P. vivax*, *P. falciparum* and *P. malariae*, mixed infections. Medium-transmission stratum: annual incidence 5–20%; no difference in incidence by age; majority of infections *P. vivax*. Low-transmission stratum: annual incidence < 5%; nearly all infections *P. vivax*

^b^Targeted population: people with a recent history of malaria and migrants from endemic areas, asymptomatic cases detected in surveys, people with enlarged spleens, people targeted for MDA but missed the treatment

^c^Intermittent preventive treatment was used for healthy individuals, mostly migrants from non-endemic areas, as chemoprophylaxis. It was also regularly performed as MDA for the entire population in high transmission stratum, active foci, or during outbreaks. Sometimes, low-dose primaquine was added for therapeutic efficacy

^d^Spraying with dichlorodiphenyltrichloroethane or hexachlorocyclohexane

Clearance of the infection source aimed to reduce transmission and prevent new cases through case management, often based on presumptive treatment and radical treatment. Radical treatment often consisted of mass drug administration (MDA) with primaquine plus another antimalarial in areas with *P. vivax,* before the transmission season for defined or entire populations (Table 1) [18, 19, 29]. MDA was also conducted using other anti-malarial drugs, such as pyrimethamine, with or without primaquine, in an evolving, adaptive way in high transmission stratum and in active foci in medium and low transmission strata during transmission seasons or outbreaks [18, 19, 29]. In China, this approach was often referred to as ‘intermittent preventive treatment’ (IPT), which is similar, but not identical to the current World Health Organization (WHO) use of the term, i.e., clearing and preventing infections in high risk target groups to reduce disease and death [30]. Vector control included indoor residual spraying (IRS) of both houses and animal sheds during transmission seasons, and treating larval breeding sites in winter and during public health campaigns [19]. This approach was important in all three strata, but the use of chemical insecticides was prioritized in areas of high transmission because of the scarcity of commodities at that time. In low-to-medium transmission strata, vector control relied on public health campaigns (Table 1).

Disruption of malaria services during the Cultural Revolution led to Province-wide outbreaks in the late 1960s and early 1970s (Fig. 3). The interventions used for outbreak containment were similar to those used for the high transmission stratum (Table 1), but were simplified to ‘three full coverage strategies’, comprising full coverage of treatment (including case management of all confirmed and clinical cases and radical treatment of the entire population), full coverage of IPT, and full coverage of IRS.

The drugs and protocols used for therapy and preventive treatment evolved with progress in drug research, development, and production (Additional file 1: Table S1) [18, 20–28]. In the 1950s, quinacrine and quinine sulphate were used, but from the 1960s, chloroquine, primaquine and pyrimethamine were manufactured in large quantities in China and were used widely within the malaria programme [31, 32]. Anti-malarial drugs were rotated during rounds of MDA to avoid or delay drug resistance [19, 29].

Implementation of the malaria control strategies had a significant impact, with the annual incidence reducing from 249.4 to 7.3 per 10,000 population between 1953 and 1967 – the largest reduction in 70-year journey towards malaria elimination in Yunnan (Fig. 3). Elimination was achieved in parts of the Province, including some high transmission areas. Menghai Basin, a township with a population of about 20,000 and a baseline prevalence of 41%, reported zero new infections in less than 5 years, after implementation of ‘three full coverage strategies’ [2, 18]. Nevertheless, consolidation of these gains was recognized as long-term process, requiring support through strong and continuous surveillance [19]. Malaria stations gradually evolved into a sanitation and anti-epidemic service covering all counties [18], and became the backbone of implementation until 2003. The extensive network of drug deliverers and sprayers, supervised by health professionals, with strong cooperation and participation by communities contributed significantly. In two counties, Jing Hong and Meng Hai, 1069 health workers representing 2.8% of the total population, were trained to implement the strategy during the 1950s–1960s, and every five households had one anti-malaria community health worker [2].

#### Targeting outbreaks to pre-elimination: 1978 to 2010.

Successful control during previous decades resulted in decreasing, further focalized transmission from the late 1970s (Fig. 2). Progress towards elimination decelerated in the early 1980s (Fig. 2), coinciding with market-oriented economic reform, which disrupted the public health function of the primary health care system [18, 33, 34]. Commodities were no longer fully funded by the Government and training of personnel was limited owing to financial constraints. These factors may explain the limited implementation of vector control and IPT, and the suboptimal performance of surveillance system [35–37]. This situation was compounded by the increase of migrant workers in the Province due to economic activities. Two regions, the Yuan River Valley and the border regions, with < 30% of the population, accounted for 90% of cases between 1980 and 1995 [2, 38]. Domestic migrant workers moving to the Yuan River Valley to engage in plantations and international migrants crossing the border for business contributed to the persistence of transmission in these areas and frequently led to outbreaks. Between 1986 and 1994, 245 outbreaks were recorded in 42 counties [2, 38]. As progress stalled, the Government resumed funding for commodities and launched a programme to monitor and evaluate the implementation of malaria interventions, with an aim to improve the performance of the system in the Yuan River Valley (1990–1997) and counties bordering the Greater Mekong Subregion (1988–1997) [2].

Large-scale MDA and spraying were no longer used, but the ‘three full coverage strategies’ remained for outbreak containment and in areas where the annual incidence was above 1%, with active foci. IRS and insecticide-treated bed nets (ITN), first with DDT, but gradually replaced by pyrethroids, were used in areas with active transmission or migrant workers to reduce malaria receptivity [39]. Therapeutic treatment of malaria cases was based on a combination of chloroquine and primaquine [40]. Chloroquine-resistant *P. falciparum* was confirmed in the 1970s, and artemisinin-based therapy gradually replaced chloroquine as first-line treatment for uncomplicated *P. falciparum* malaria (Additional file 1: Table S1) [18, 20–28, 31, 38]. Radical treatment before transmission seasons targeted people who had contracted malaria in the previous year. Most cases were confirmed with a laboratory test before treatment, but in remote areas with limited diagnostic capacity, presumptive treatment was provided to patients with fever. IPT remained an important strategy [41], targeted primarily at migrants travelling to endemic countries to reduce morbidity, although implementation was operationally challenging. Case and focus investigations began gradually in areas with an annual incidence < 1/1000, though a specific timeline was not specified. Focus investigation and response included reactive case detection, focal MDA, and reactive vector control.

**Fig. 4:**
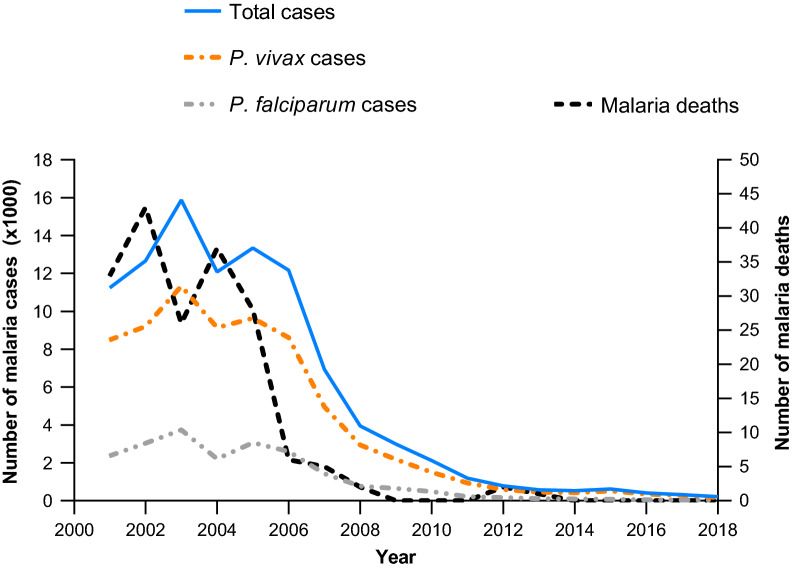
Malaria cases and deaths in Yunnan 2001–2018 by species

After the outbreak of SARS in 2003, malaria case detection increased, extended with international funding [42]. The coverage and quality of diagnosis, treatment and vector control were further improved by extensive training of front-line health workers, especially in rural villages [42]. Progress towards malaria elimination was accelerated, and the case load dropped significantly between 2003 and 2010 (Fig. 4). The numbers of cases of both falciparum and vivax malaria decreased simultaneously (Fig. 4), suggesting that the control measures impacted both species, including both long and short incubation *P. vivax* sub-species.

#### Pre-elimination to elimination: 2010 to 2018

**Fig. 5:**
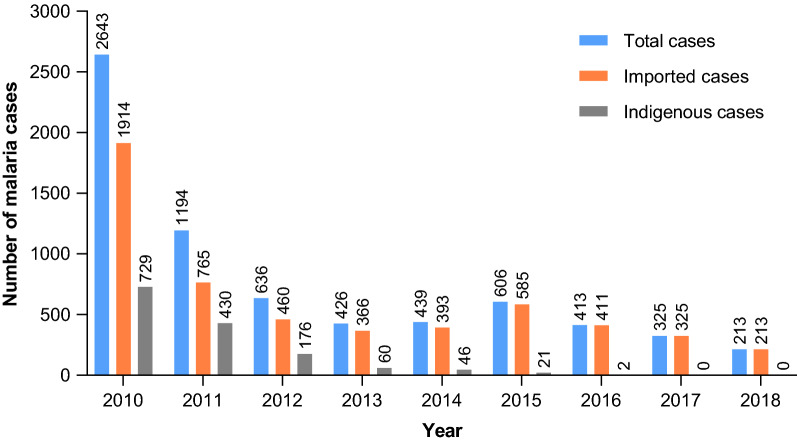
Malaria cases in Yunnan by source of infection 2010–2018

When China launched its national malaria elimination action plan in 2010 [21], there were 729 indigenous cases (*P. falciparum* and *P. vivax*) in 39 of the 129 counties of Yunnan (Fig. 5). Although this was far fewer cases compared with the malaria burden even ten years before, it was not until 2017 that zero indigenous cases were reported.

**Fig. 6:**
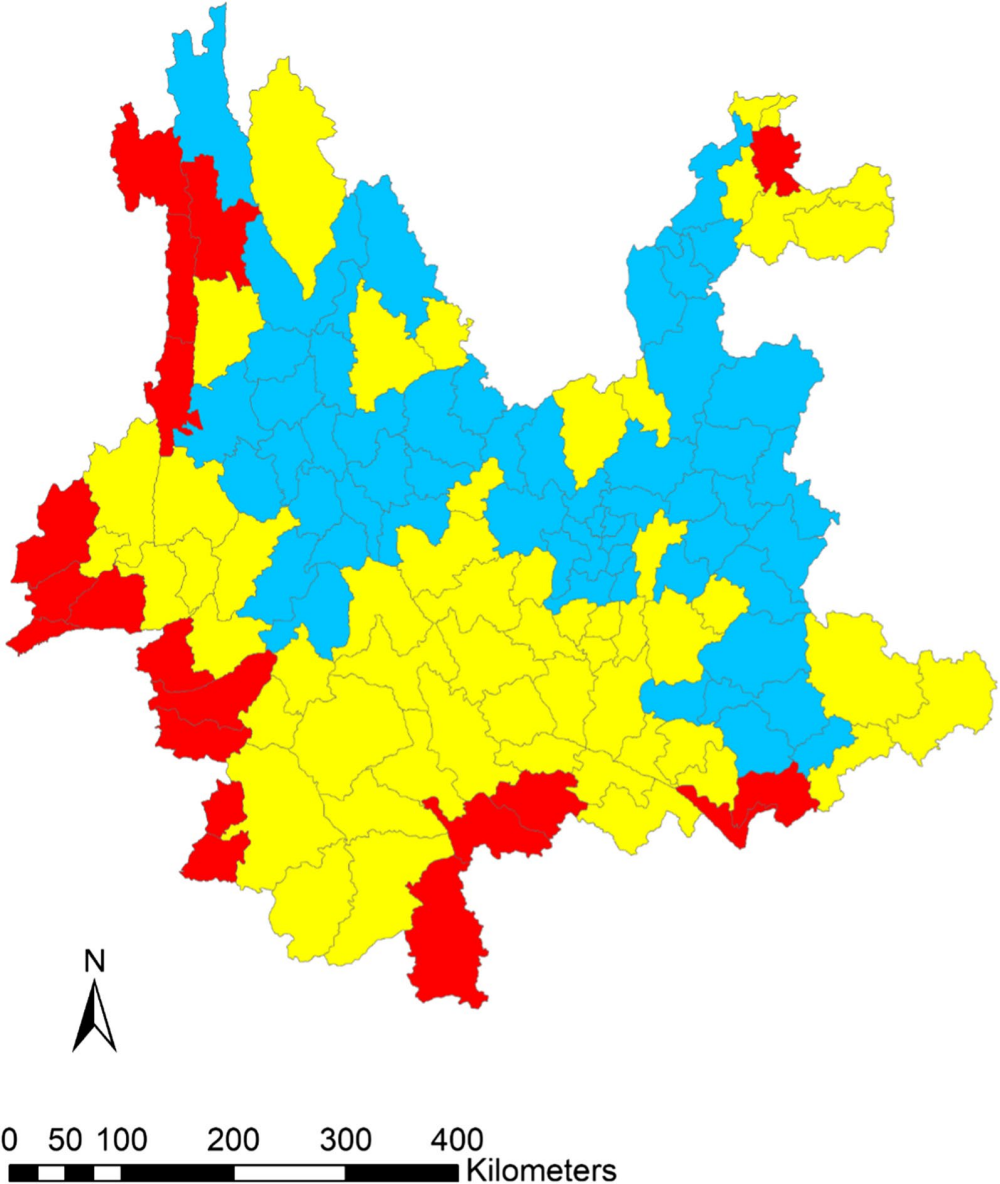
Map of Yunnan showing stratification for malaria elimination in 2010. Type I (19 counties, red): indigenous cases were detected every year over the previous three consecutive years and the annual incidence was > 1/10,000; Type II (55 counties, yellow): indigenous cases were detected at least once over the previous three consecutive years, and the annual incidence was < 1/10,000; Type III (55 counties, blue): no indigenous cases detected

Counties were stratified into three categories (I, II, and III) based on the occurrence of indigenous cases and incidence (Fig. 6). Case management practice was changed from presumptive to laboratory-confirmed cases. Routine vector control was used in strata I and II where foci were not cleared. Also, the ‘1–3-7′ approach was introduced as the combined standard operating procedure for surveillance and response, specifying case reporting within 1 day after diagnosis, case investigation within 3 days, and focus investigation and action within 7 days [43–48]. Depending on the type of focus, the response could include reactive case detection, with recent fever as an indicator for testing a blood sample, focal MDA, vector control (IRS or ITNs) and health education [21]. In remote areas, case and focus investigation and response (3 and 7) were sometimes combined in one visit. A mid-term assessment showed that > 99.8% of cases were reported within 24 h and 99.4% of cases were investigated within 3 days [42]. Additionally, proactive case detection was conducted during transmission seasons in villages with migrants and efficient vectors.

The programme collaborated with other departments, including customs, to raise awareness and improve the health seeking behaviour of migrants crossing the international border. Overall, Yunnan adopted a holistic approach for malaria elimination, monitoring and evaluating a spectrum of metrics, including the timeliness of case notification, case investigation, focus investigation, staff training, health education, and multi-sectoral collaboration.

### Border malaria

#### The multifaceted nature of border malaria

The long, porous Yunnan international border provides no natural barriers and numerous informal crossing points. Thirteen ethnic groups have straddled the border for generations, and economic ties between Yunnan and neighbouring countries date back to ancient times. Borders exist only as a political construct, with people crossing daily for business, to visit family, for schooling, or medical care. Although the two sides of the border have the same ecological environment, with similar malaria vectors [49, 50], surveys and studies reveal a significant gradient in malaria transmission, with the malaria prevalence in migrants from neighbouring countries five to seven times higher than that of residents of Yunnan border counties [51, 52].

**Fig. 7:**
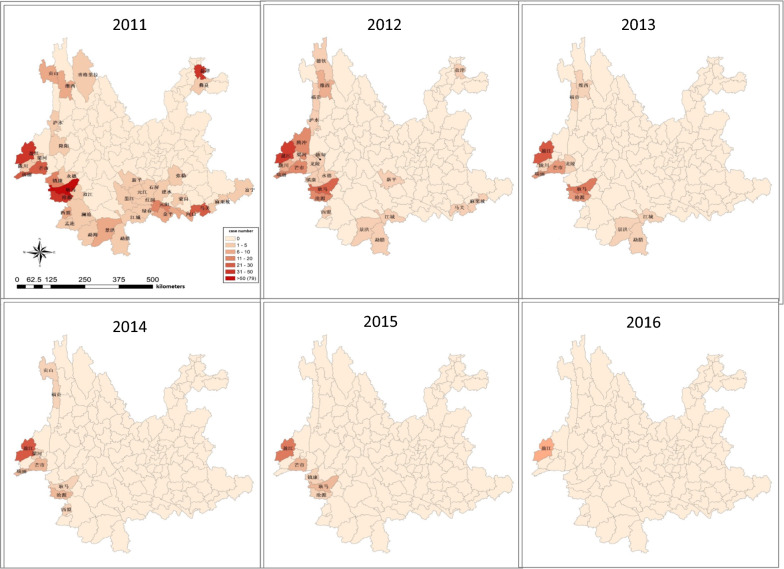
Counties with indigenous cases in Yunnan (2011–2016)

From the start of the national elimination programme, malaria cases gradually concentrated along the border between Yunnan and Myanmar (Fig. 7), with the final focus in Yingjiang county (Fig. 7). Border areas represent one of the greatest challenges to malaria elimination in Yunnan for several reasons: malaria endemicity was historically high [2, 19]; poverty is more prevalent in border areas and is correlated with malaria incidence in Yunnan [23]; populations can live in physically remote, inaccessible regions [24]; and high illiteracy rates and inappropriate health-seeking behaviour among ethnic groups contribute to a higher malaria prevalence [25–28, 53]. Since 2001, > 90% of malaria cases in Yunnan have been detected in border regions [54], most of which were imported from countries in the Greater Mekong Subregion. For example, the border county of Tengchong reported > 2000 imported cases in 2006 [55].

**Fig. 8:**
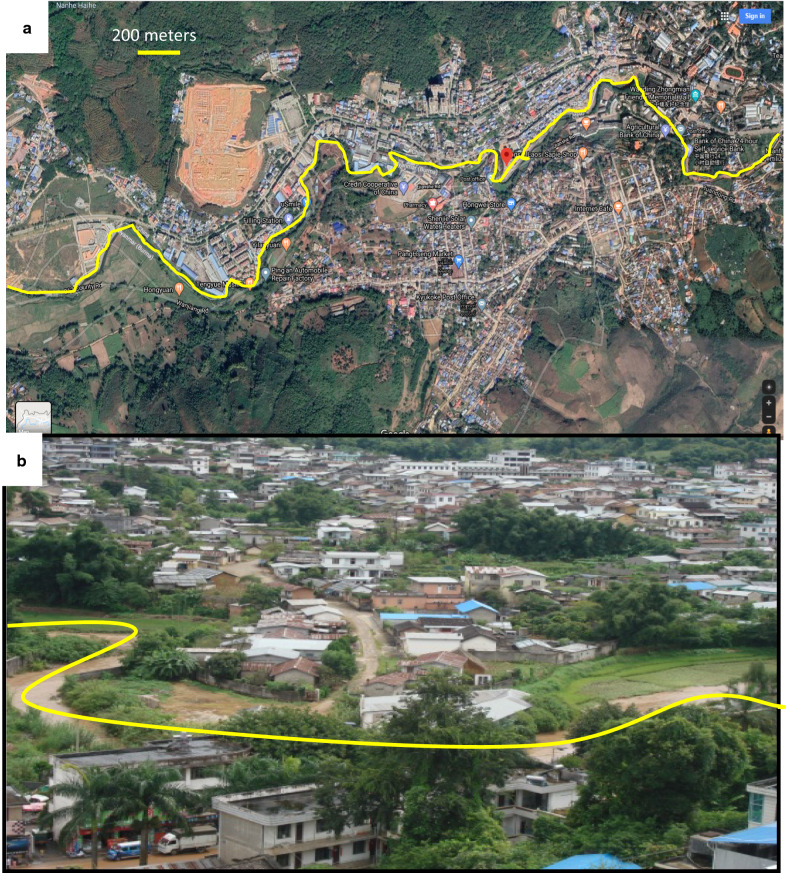
Villages that straddle the Yunnan–Myanmar international border**.** **a** Bird’s eye view of twin towns Wanding (Ruili, Yunnan)—Pang Hseng (Myanmar). **b** Nabang (Yingjiang, Yunnan)—Laiza (Myanmar)

The case load itself represented an operational challenge for elimination. In counties with high cases loads, 81.2% of cases be completed within 3 days compared with 94% in those with a lower case load [47, 56]. This was probably because timeliness and intensity of surveillance and response requirements could not be fully met. The challenge was not limited to the management of imported cases and the risk of onward transmission. Outbreaks on the other side of the border could quickly spread to Yunnan when people hurried back for health care [57]. One study showed that the numbers of imported and local cases in Yunnan border counties were correlated with malaria prevalence in Myanmar border regions [58]. Furthermore, some border villages straddle international borders, effectively representing shared foci (Fig. 8). These villages present an additional challenge for transmission interruption as interventions were difficult to implement equally throughout the shared focus. The county of Yingjiang, where the last foci were found in the Province (Fig. 7), reported 17 local cases in 2014, 15 of which were < 200 m from communities in Myanmar [58].

#### Strategies for eliminating border malaria

Pilot studies showed that chemoprophylaxis targeting migrants crossing international borders could reduce the monthly malaria incidence from > 10 to < 1% [58, 59]. A malaria kit containing chemoprophylaxis, mosquito repellents, and a health information leaflet was distributed at border health facilities and immigration offices [2]. The 1-3-7 strategy remained the primary tool for malaria elimination in border areas. However, in villages < 2 km from the border (estimated to be the flying range of 80% of mosquitoes, data of the Yunnan programme), all households were covered with IRS or long-lasting insecticidal bed nets. These strategies, with cross-border collaboration, were known as the ‘three defensive lines strategies for border malaria’ (Table 2) [2]. Key components of these strategies were good surveillance and response at the lowest level, and free provision of malaria diagnosis and treatment, regardless of nationality. Front-line health workers in border health facilities were trained more frequently and received more frequent monitoring and supervisory visits from the programme. Two additional temporary laboratory technicians were recruited in 2015 to support case detection and distribution of malaria kits in 68 health facilities situated along the international border [60].

**Table 2:** Three defensive lines strategies to manage border malaria in Yunnan

Line	Scope	Objectives	Measure
1st	Border counties, including townships and villages	Timely detection and response for every case according to the requirements of ‘1–3-7’	Strengthen surveillance and response capacity Improve the quality and capacity for case detection and case management in village and township hospitals by training, monitoring and supervision
2nd	International borderline	Improve case detection effectiveness; ensure all suspected cases are tested Reduce importation from neighbouring countries Reduce receptivity in border villages	Register and monitor migrants, in collaboration with immigration offices Distribute malaria kits at border health facilities and immigration offices Increase human resources in laboratories in border health facilities Detect cases proactively in border villages with migrants Conduct vector control in border villages
3rd	Myanmar border regions	Reduce incidence of malaria in Myanmar border region and importation to Yunnan	Support capacity building in Myanmar border regions through training and technical support Support outbreak response Provide commodities as required Exchange information

#### Cross-border collaboration

Funded by the Chinese Government, from 2005 Yunnan established cross-border collaboration on malaria, dengue, and other communicable diseases with neighbouring countries. Under the agreements, Yunnan has promoted capacity building by providing commodities, training, and support for the establishment of laboratories and other facilities (Table 2) [61]. Case and focus investigation and vector control have been synchronized in shared villages and twin towns whenever possible. Joint advocacy activities are conducted each year with countries in the Greater Mekong Subregion on World Malaria Day.

Of the three neighbouring countries, Myanmar has the highest malaria burden and the longest border with Yunnan (> 2000 km). Large-scale implementation of interventions in border areas of Myanmar began only in 2008 [62], with access to health services limited because of restricted socioeconomic development, remoteness, and poverty. The internal conflict in Myanmar, started in 1948 when the country declared independence, has resulted in large numbers of internally displaced persons fleeing to areas close to international borders, including those bordering Yunnan [63]. These areas are under the control of several armed ethnic groups, each with a different status under the ceasefire agreement. Cross-border collaboration is thus challenging.

A project for malaria diagnosis and treatment, use of ITNs, and health education targeted to both migrant Chinese workers and local border residents in Myanmar, supported by the World Health Organization and funded by a Global Fund–China grant, was implemented from 2006 to 2013 [64]. In this project, Health Poverty Action (HPA, formerly known as Health Unlimited), an NGO headquartered in the United Kingdom, established an antimalarial network in the politically complex environment of the Myanmar border areas. The network comprised technicians for microscopic examination, outreach teams, private clinics, village malaria workers and migrant malaria workers. These services provided malaria diagnosis and treatment, not only to residents along the Myanmar border, including remote areas, but also to Chinese migrant workers in Myanmar. The malaria burden in these regions was reduced by 89% [62], and the 19 counties in Yunnan that border Myanmar achieved a 95% reduction. The most significant reduction in the number of malaria cases in Yunnan since 2001 was observed between 2006 and 2008 (Fig. 4), coinciding with implementation of this cross-border project. The strong correlation between the reductions in malaria in Yunnan border counties and in Myanmar border regions [58], suggests that the reduction of malaria in Myanmar border regions promoted malaria elimination in Yunnan.

### Challenges for malaria elimination and preventing re-establishment

#### Complexity of ecological features and malaria epidemiology

Although national policies and operational manuals have provided clear guidance, the complex malaria epidemiology of Yunnan has complicated the selection of strategies for malaria elimination (Table 1, Additional file 1: Table S1) [18, 20–28]. For example, one county used infection source clearance as the main technical strategy based on guidance from national strategy [40], plus an entomological study which reported *An. sinensis* as a vector in one village. However, this approach was shown to be inappropriate when outbreaks occurred in several villages a few years later [33]. Similarly, one to two rounds of IRS was suggested for areas with *An. minimus* based on national policies [40], but three rounds were necessary in some places when the transmission seasons were long. Thus, operational studies to assess intervention effectiveness and the flexibility to adjust activities to local conditions were important components of the strategy.

An even greater challenge is estimating malaria receptivity, an important but elusive factor determining the potential for transmission. For example, altitudes > 1700 m were thought to be at very low risk of malaria, but transmission of *P. falciparum* and *P. vivax* by *Anopheles kunmingensis* was re-established in the 1990s at these altitudes, after recording zero transmission since the 1950s [65]. Province-wide, malaria receptivity is probably declining, because of improved housing conditions, the destruction of breeding sites caused by changing land use, and a strong health system. *An. kunmingensis* used to be the primary vector in Tengchong but has been rarely found there recently [16, 66]. However, *An. minimus* remains abundant in the border county of Yingjiang [67], and the anopheline community remains complex and stable during the entire epidemic season in low elevation areas [68]. Moreover, studies showed that deforestation enhanced the survival of *An. minimus* larvae and adults in this area [69, 70]. These findings support the relatively high malaria receptivity documented in unpublished grey literature.

#### High-risk groups

Studies indicated that minority groups in Yunnan were at high risk for malaria [71, 72]. Such groups happened to live in hard to reach areas with limited access of health care [24]. Their poorer socioeconomic status created barriers to the use of bed nets or other preventive measures [28, 58, 73]. Lack of knowledge on malaria or the benefits of preventive intervention was also a factor [74], though health education, such as behaviour change communication strategies, could improve bed net use from 16.1 to 87.9% [75].

Mobile and migrant populations have been the major high-risk groups for malaria in Yunnan [51, 72, 76, 77]. Domestic migrants, travelling from non-transmission provinces or areas to endemic areas in Yunnan for plantation or construction work, were at high risk because of their low immunity to malaria [2, 78, 79]. Similarly, people from high altitude areas were at risk of malaria when they went down to mid-mountain areas, basins and river valleys for agriculture. International migrants at risk of malaria included Yunnan workers who crossed the border for business and returned from the neighbouring countries, legal or illegal nationals from neighbouring countries, and refugees from conflict [24]. One study found that travel to lowland and foothill or mid-hill hyperendemic areas in Myanmar, especially areas along the waterside, was a high-risk factor for malaria [76, 77]. Interestingly, forest goers, unlike those in South-East Asia, were not particularly at risk of malaria [76]. International migrants, particularly Yunnan migrant workers returning from endemic areas, continue to be a high*-*risk group.

#### Challenges to maintaining malaria-free status

Border malaria remains one of the biggest threats for the re-establishment of malaria transmission. There is strong evidence that imported parasites from neighbouring countries can be transmitted in Yunnan [2, 65]. The risk of the re-establishment of transmission is significant with continuous importation. In concert with local governments, the malaria programme has been able to actively collaborate with other sectors to target surveillance activities to the migrant population. However, this requires continued government support to maintain a strong surveillance system to manage the risk of re-establishment of transmission [2]. Malaria elimination in border areas of neighbouring countries, i.e. Myanmar, is unlikely to be achieved in near future. The current implementation of malaria services by HPA in the Myanmar border regions relied on international funding, which might not be sustainable. The complex political environment is unlikely to be resolved in the near future and cross-border collaboration will remain challenging.

As malaria is eliminated, vigilance in the general health service naturally declines as malaria becomes a ‘rare’ disease. Indeed, comparisons of quality assurance results across Yunnan have shown that prefectures that saw more malaria cases performed better in providing quality assured diagnosis [80]. A stable primary health care network and a professional malaria team is a fundamental requirement; a lesson learned from the history of malaria elimination in Yunnan. The primary health care system has been significantly improved following its reform since 2003. Nevertheless, challenges remain, including frequent staff turnover [81], and the CDC system, created in 2003 with a goal to strengthen the public health service, struggles to maintain the necessary levels of technical expertise. Between 2003 and 2018, the number of personnel and professionals reduced by 10.6 and 12.2%, respectively, for various reasons, including low salary [82].

## Conclusion

Seven decades of efforts in Yunnan have led to the successful interruption of malaria transmission in Yunnan. The malaria elimination story in Yunnan has shown the importance of strong and continuous political commitment from governments at all levels, which must be translated into the resources needed to ensure effective implementation: 1) universal malaria care to the entire population, regardless of status; 2) a stable primary health care system to ensure health service delivery; 3) strategies and interventions developed and selected based on the best knowledge, data available, experiences, and the local context; 4) data-driven decision making to achieve impact and advance elimination in a resource constrained environment; 5) a robust surveillance system which performs effectively at all levels; 6) extensive participation by communities for successful implementation of interventions; 7) recognising the challenge of border malaria early, with more resources diverted to border areas. Yunnan will have to take all the experiences and lessons learned from elimination and manage the challenges for prevention re-establishment of transmission, including border malaria, to sustain its malaria-free status.

## Supplementary Information

**Additional file 1**: **Table S1**. Antimalarial drugs used in Yunnan 1950s to 2016 [18, 20-28].

## Data Availability

All data reviewed are included in this published article and its supplementary information files.

## Abbreviations

CDC: Chinese Center for Diseases Control and Prevention
Global Fund: Global Fund to Fight AIDS, Tuberculosis and Malaria
HPA: Health Poverty Action
IPT: Intermittent preventive treatment
IRS: Indoor residual spraying
ITN: Insecticide-treated bed nets
NIPD: National Institute of Parasitic Diseases
MDA: Mass drug administration
YIPD: Yunnan Institute of Parasitic Diseases
