# Supplementary material for: Seven decades towards malaria elimination in Yunnan, China
Source: Malar J. 2021 Mar 12;20:147. doi: 10.1186/s12936-021-03672-8 (PMC7953382; doi:10.1186/s12936-021-03672-8)
Supplement: Supplementary file 1 — Additional file 1: Table S1. Antimalarial drugs used in Yunnan 1950s to 2016 [18, 20-28]. [file 12936_2021_3672_MOESM1_ESM.docx]

**Table S1. Antimalarial drugs used in Yunnan 1950s to 2016.^a^**

| **Era** | **Case management: *P. vivax*, *P. ovale*, *P. malariae*** | **Case management: *P. falciparum*^b^** | **Radical treatment: *P. vivax*** | **Intermittent preventive treatment or intermittent treatment** |
| --- | --- | --- | --- | --- |
| 1950s | Quinacrine, quinine sulphate | Quinacrine, quinine sulphate |  | Paludrine, quinacrine, quinine sulphate, pamaquine |
| 1960s–1970s | - **CQ + PQ 8-day treatment**   CQ: 1200 mg (600 mg day 1, 300 mg days 2 and 3); PQ: 180 mg (22.5 mg/day x 8 days)   - **PYR + PQ 8-day treatment**   PYR: 1500 mg (600 mg day 1, 450 mg days 2 and 3); PQ: 180 mg (22.5 mg/day x 8 days)   - **CQ + PQ 4-day double treatment**   CQ: 1200 mg (600 mg each days 1 and 2, 300 mg day 3); PQ: 180 mg (22.5 mg/day x 4 days, after 7 days to 1 month; 22.5 mg/day x 4 days)   - **Intermittent treatment**   Every week or every 10 days; CQ: 300 mg + PQ: 30 mg/week x 8 weeks   - **Quinine sulphate**   1.5 g per day x 7–10 days | - **CQ + PQ 4-day treatment**   CQ: 1500 mg (600 mg day 1, 450 mg days 2 and 3); PQ: 90 mg (22.5 mg/day x 4 days)   - **CQ + PQ 3-day treatment**   CQ: 1500 mg (600 mg day 1, 450 mg days 2 and 3); PQ: 67.5 mg (22.5 mg/day x 3 days)   - **Treatment for CQ resistance**   PIP or Fang San^e^ + PQ or quinine sulphate | - **PYR + PQ**   PQ: 180 mg (22.5 mg/day x 8 days) + single dose of PYR (50 mg) before or after   - **CQ + PQ 8-day treatment**   CQ: 1500 mg (600 mg day 1, 450 mg days 2 and 3); PQ: 180 mg (22.5 mg/day x 8 days)   - **CQ + PQ 4-day double treatment**   CQ: 1200 mg (600 mg days 1 and 2, 300 mg day 3); PQ: 180 mg (22.5 mg/day x 4 days; 22.5 mg/day x 4 days after 7 days to 1 month) | - **CQ:** 300 mg every 10 days for 3 months - **PYR:** 50 mg every 15 days - **Fang Yi^c^:** 1 tablet every 10–15 days - **Fang Er^d^:** 2 tablets every 10–15 days - **Fang San^e^:** 4 tablets every 25–30 days - **PYR + PQ**   PYR: 50 mg; PQ: 30 mg, every 7–10 days, repeated at least 8 times   - **CQ + PQ**   CQ: 300 mg; PQ: 30 mg every 7–10 days, repeated at least 8 times |
|  | - **CQ + PQ 8-day treatment**   CQ: 1200 mg or 1500 mg (600 mg day 1, 300 mg or 450 mg days 2 and 3); PQ: 180 mg (22.5 mg/day x 8 days)   - **CQ + PQ 4-day double treatment**   CQ: 1200 mg (600 mg each days 1 and 2, 300 mg day 3); PQ: 180 mg (22.5 mg/day x 4 days, after 7 days to 1 month; 22.5 mg/day x 4 days) | - **CQ+PQ 3-day treatment**   CQ: 1200 or 1500 mg (600 mg day 1, 300 mg or 450 mg days 2 and 3); PQ: 67.5 mg   - **Treatment for CQ resistance**   PIP, PYN or AM, AS, DHA | As above | - **Fang Er^d^:** 3 tablets + 30 mg PQ, every 10 days - **Fang San^e^:** 3 tablets + 30 mg PQ, every 20 days - **CQ:** In areas endemic for *P. vivax* - **PIP:** 0.6 g every month - **PYR + PQ**   PYR: 50 mg; PQ: 22.5 mg, every 7–10 days, repeated at least 8 times |
| 2002 | - **CQ + PQ 8-day treatment**   CQ: 1200 mg (600 mg day 1, 300 mg days 2 and 3); PQ: 180 mg (22.5 mg/day x 8 days)   - **CQ + PQ 4-day double treatment**   CQ: 1200 mg (600 mg each days 1 and 2, 300 mg day 3); PQ: 180 mg (22.5 mg/day x 4 days, after 7 days to 1 month; 22.5 mg/day x 4 days) | - **CQ + PQ 3-day treatment**   CQ: 1500 mg (600 mg day 1, 450 mg days 2 and 3); PQ: 22.5 mg/day x 2 days   - **PIP + PQ**   PIP: 1500 mg (600 mg day 1, 450 mg days 2 and 3) PQ: 22.5 mg/day x 2 days   - **Treatment for CQ resistance**   AS, AM, DHA, PYN, or PYN + any of the following three drugs: DHA or AM or AS; PQ added to all treatments | - **CQ + PQ 8-days treatment**   CQ: 1500 mg (600 mg day 1, 450 mg days 2 and 3); PQ: 180 mg (22.5 mg/day x 8 days) | - **PIP:** 600 mg for migrants every month (*P. falciparum* and *P. vivax*) - **CQ:** (only *P. vivax*) |
| 2016 | - **CQ + PQ 8-day treatment**   CQ: 1200 mg or 1500 mg (600 mg day 1, 300 mg (or 450 mg) days 2 and 3); PQ: 180 mg (22.5 mg/day x 8 days)   - **ACT + PQ** | - **PYN:** Total dose, 1200 mg (600 mg day 1, 300 mg days 2 and 3) - **ACT** | - **PQ:** 180 mg (22.5 mg/day x 8 days) | - **PIP:** 600 mg for migrants every month (*P. falciparum* and *P. vivax*); ≤ 4 months - **CQ:** In areas endemic for *P. vivax* |

ACT, artemisinin-based combination therapy; AM, artemether; AS, artesunate; CQ, chloroquine; DHA, dihydroartemisinin; PIP, piperaquine; PQ, primaquine; PYN, pyronaridine; PYR, pyrimethamine.

^a^ All treatment and dosages listed only for adults.

^b^ Treatment for *P. falciparum* infection is for uncomplicated malaria.

^c^ Fang Yi: 1st-generation compound for malaria prevention. Each contained 50 mg PYR and dapsone; not in use since 1970s.

^d^ Fang Er: 2nd-generation compound for malaria prevention. Each tablet contained 250 mg sulphadoxine + 17.5 mg PYR.

^e^ Fang San: 3rd-generation compound for malaria prevention. Each tablet contained 250 mg PIP + 50 mg sulphadoxine.
